# Late Vitamin K Deficiency Bleeding Presenting as Subacute Subdural Hemorrhage in an Exclusively Breastfed Infant: A Preventable Life‐Threatening Condition—A Case Report

**DOI:** 10.1002/ccr3.73365

**Published:** 2026-08-23

**Authors:** Yasir Khalif Ali, Mohamed Nur Ali, Fardowsa Hassan Ahmed, Farah Ali Ahmed, Farah Abdullahi Ismail, Ibrahim Mohamed Hirsi, Said Abdirahman Ahmed, Mohamed Mukhtar Mohamed

**Affiliations:** ^1^ Department of Pediatrics Mogadishu Somali Turkiye Training and Research Hospital Mogadishu Somalia; ^2^ Faculty of Medicine & Health Sciences Hormuud University Mogadishu Somalia; ^3^ Department of Cardiology Mogadishu Somali Turkiye Training and Research Hospital Mogadishu Somalia; ^4^ Department of Emergency Medicine Mogadishu Somali Turkiye Training and Research Hospital Mogadishu Somalia

**Keywords:** case report, exclusively breastfed infant, intracranial hemorrhage, late VKDB, neonatal vitamin K prophylaxis, subdural hemorrhage, vitamin K deficiency bleeding

## Abstract

Late vitamin K deficiency bleeding should be considered in exclusively breastfed infants presenting with seizures, pallor, or unexplained bleeding, particularly when vitamin K prophylaxis was not administered at birth. Early diagnosis and prompt vitamin K administration can rapidly correct coagulopathy and prevent life‐threatening intracranial hemorrhage.

## 1 Introduction

Vitamin K plays a crucial role in normal blood coagulation through the activation of several clotting factors synthesized in the liver. Newborn infants are particularly vulnerable to vitamin K deficiency because placental transfer is limited, hepatic vitamin K stores are low at birth, the concentration of vitamin K in human breast milk is relatively low, and intestinal bacterial synthesis remains immature during early infancy [1, 2].

Vitamin K deficiency bleeding (VKDB) is an acquired hemorrhagic disorder that results from inadequate activity of vitamin K–dependent coagulation factors. Clinical manifestations range from mild cutaneous bleeding to severe hemorrhage involving the gastrointestinal tract, mucosal surfaces, or central nervous system [3]. Based on age at presentation, VKDB is categorized as early, classic, or late disease. Late VKDB typically develops between 2 weeks and 6 months of age and occurs most commonly among exclusively breastfed infants who did not receive vitamin K prophylaxis at birth. Infants with underlying cholestatic or malabsorptive disorders are also at increased risk [4].

Intracranial hemorrhage represents the most devastating complication of late VKDB and is associated with substantial mortality and long‐term neurological disability. Common presenting features include seizures, irritability, poor feeding, pallor, altered consciousness, and bulging fontanelle [5]. Because routine neonatal vitamin K administration is highly effective in preventing this condition, cases of late VKDB are largely avoidable. Universal neonatal vitamin K prophylaxis remains a critical public health intervention. Here, we report a 2‐month‐old exclusively breastfed male infant who was born at home without vitamin K prophylaxis and presented with generalized convulsion due to late VKDB complicated by subacute subdural hemorrhage.

## 2 Case History and Examination

A 2‐month‐old male infant, born at home to non‐consanguineous parents, was brought to the emergency department after a single episode of generalized afebrile convulsion. In the emergency room, the patient developed profuse bleeding following intravenous cannulation. According to his parents, he had been healthy until 24 h before admission, when he became increasingly irritable, appeared pale, and developed decreased oral intake.

There was no history of trauma, surgery, medication use, or previous bleeding episodes. Family history was unremarkable for bleeding disorders or similar conditions. Nutritional history revealed exclusive breastfeeding. Regarding prenatal and birth history, the mother had not attended routine antenatal care visits and had not taken any medications during pregnancy. The pregnancy and spontaneous vaginal delivery were otherwise uncomplicated. The infant did not receive vitamin K prophylaxis at birth.

On physical examination, the infant appeared severely ill, lethargic, and markedly pale, with no jaundice. His vital signs showed a heart rate of 60 beats/min, irregular respiratory rate of 30 breaths/min, body temperature of 36°C, and random blood glucose level of 110 mg/dL. Oozing of blood was noted from previous peripheral intravenous cannulation sites. Abdominal and cardiovascular examinations were unremarkable. Neurological examination revealed a bulging, nonpulsatile anterior fontanel. No facial asymmetry, abnormal posturing, or pupillary abnormalities were observed.

## 3 Differential Diagnosis, Investigations, and Treatment

Differential diagnoses considered included congenital coagulation disorders, disseminated intravascular coagulation, liver disease‐associated coagulopathy, sepsis‐related bleeding, and non‐accidental trauma. Inherited coagulation disorders were considered less likely because there was no family history of bleeding disorders, no previous bleeding episodes, and the coagulopathy corrected rapidly after vitamin K administration. Liver disease‐associated coagulopathy was also considered; however, there was no clinical jaundice, no hepatosplenomegaly, and no evidence of chronic liver disease or malabsorption during admission. Disseminated intravascular coagulation was unlikely because the platelet count and fibrinogen level were preserved, and there was no clinical evidence of severe sepsis. Non‐accidental trauma was considered because of the intracranial hemorrhage; however, there was no reported history of trauma, no external injuries were identified on examination, and the overall clinical and laboratory profile was more consistent with late VKDB. The absence of neonatal vitamin K prophylaxis, exclusive breastfeeding, markedly prolonged PT/aPTT/INR with normal platelet count, and rapid correction after vitamin K and plasma therapy strongly supported the diagnosis of late vitamin K deficiency bleeding.

**FIGURE 1:**
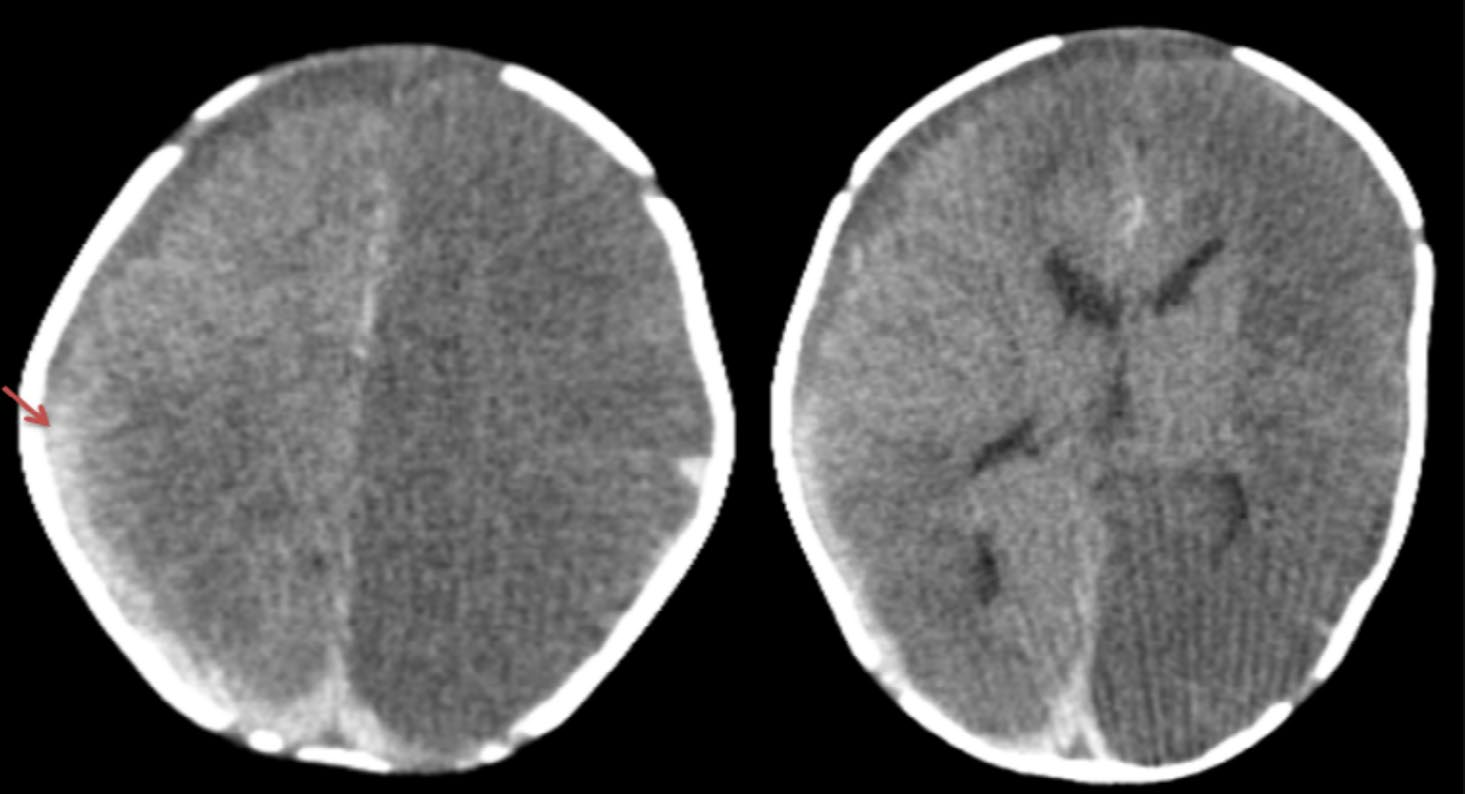
Non‐contrast axial computed tomography of the brain demonstrating diffuse hypoxic–ischemic cerebral changes with a mild right‐sided subacute subdural hematoma.

Initial laboratory investigations are summarized in Table 1. The results demonstrated severe anemia with a normal platelet count, mildly elevated total and direct bilirubin levels, and markedly deranged coagulation parameters. Initial urinalysis also revealed hematuria. Non‐contrast cranial computed tomography showed diffuse bilateral cortical–subcortical hypodensity with significant effacement of the cerebral sulci and cisterns, suggestive of cerebral hypoxic changes. Minimal fluid‐level hypodensities were also seen in the right subdural region, measuring approximately 5 mm, consistent with a subacute subdural hematoma (Figure 1).

**TABLE 1:** Initial and discharge laboratory investigations.

Parameter	At admission	At discharge	Reference range
Hemoglobin (Hgb)	3.1 g/dL	11 g/dL	10.5–14 g/dL
Hematocrit (Hct)	9.8%	35%	33%–42%
Platelet count (PLT)	233,000/μL	400,000/μL	150,000–450,000/μL
INR	4.0	0.9	0.8–1.2
aPTT	Prolonged beyond measurable range	32 s	25–40 s
PT	120 s	13 s	10–16 s
Fibrinogen	270 mg/dL	Not measured	200–400 mg/dL
D‐dimer	Not measured	Not measured	< 0.5 μg/mL
AST	75 U/L	35 U/L	0–31 U/L
ALT	30 U/L	28 U/L	0–45 U/L
Total bilirubin	1.84 mg/dL	Not measured	0.30–1.10 mg/dL
Direct bilirubin	1.36 mg/dL	Not measured	0.01–0.40 mg/dL

A diagnosis of late‐onset vitamin K deficiency bleeding (VKDB) was made based on the clinical presentation, absence of vitamin K prophylaxis at birth, exclusive breastfeeding, bleeding manifestations, normal platelet count, and markedly prolonged coagulation parameters. In the emergency department, the patient was immediately intubated, stabilized, and transferred to the pediatric intensive care unit (PICU).

Management included three intravenous doses of vitamin K, fresh frozen plasma at 15 mL/kg, and two transfusions of packed red blood cells at 20 mL/kg each. Within less than 8 h, the coagulation profile and hematologic parameters showed marked improvement, and active bleeding resolved clinically. The patient remained intubated and was managed with a midazolam infusion for sedation and seizure control. Mannitol infusion at 0.5 g/kg was administered for 2 days to reduce intracranial pressure.

## 4 Outcome and Follow‐Up

Following administration of intravenous vitamin K, fresh frozen plasma, and packed red blood cell transfusions, the patient's bleeding manifestations resolved clinically and coagulation parameters improved markedly within 8 h. The INR decreased from 4.0 at admission to 0.9 by discharge, PT improved from 120 s to 13 s, and aPTT normalized from being beyond the measurable range to 32 s. Hemoglobin improved from 3.1 g/dL at admission to 11 g/dL at discharge following transfusion support. He remained in the pediatric intensive care unit for ongoing neurological monitoring and supportive management. No further seizures or bleeding episodes occurred during hospitalization.

After a two‐week hospital stay, the infant was discharged in stable condition. At follow‐up 1 week later, he demonstrated appropriate feeding, good neck control, and age‐appropriate developmental progress. No focal neurological deficits, recurrent seizures, or bleeding episodes were identified during early follow‐up.

## 5 Discussion

Late vitamin K deficiency bleeding (VKDB) remains an important and preventable cause of serious bleeding in young infants, particularly in regions where neonatal vitamin K prophylaxis is not universally administered. The condition occurs most frequently in exclusively breastfed infants because human breast milk contains relatively low concentrations of vitamin K compared with formula feeds. Additional risk factors include home delivery, parental refusal of prophylaxis, cholestatic liver disease, and disorders associated with malabsorption of fat‐soluble vitamins [1, 2, 4].

Intracranial hemorrhage is the most severe manifestation of late VKDB and represents a major cause of mortality and long‐term neurological disability among affected infants. Previous studies have reported mortality rates ranging from 20% to 50%, with survivors at risk of developmental delay, epilepsy, and permanent neurological impairment [5, 6]. Clinical presentation is often nonspecific and may include poor feeding, irritability, pallor, vomiting, seizures, altered consciousness, or bulging fontanelle. Because these findings may mimic central nervous system infection, trauma, or congenital bleeding disorders, a high index of suspicion is required, particularly in infants who did not receive vitamin K prophylaxis at birth [4, 5].

Our patient demonstrated several classical features of severe late VKDB, including exclusive breastfeeding, absence of neonatal vitamin K administration, severe anemia, bleeding from venipuncture sites, markedly prolonged coagulation parameters, and intracranial hemorrhage. Neuroimaging revealed a right‐sided subacute subdural hematoma accompanied by diffuse hypoxic–ischemic cerebral changes. Although mild direct hyperbilirubinemia was present, no clinical or laboratory evidence of underlying chronic liver disease, cholestasis, or malabsorption was identified during hospitalization. The combination of a normal platelet count, preserved fibrinogen level, markedly prolonged PT and INR, and rapid correction following vitamin K administration strongly supported the diagnosis of late VKDB [7].

Recent reports continue to demonstrate that late VKDB may present catastrophically with intracranial hemorrhage in infants who did not receive vitamin K prophylaxis. Choudhary et al. reported acute‐on‐chronic subdural hemorrhage due to late vitamin K deficiency, highlighting the diagnostic importance of severe coagulopathy with preserved platelet count in an infant with intracranial bleeding [8]. More recently, Doğan et al. described four fatal cases of late‐onset VKDB with clinical‐autopsy correlation, emphasizing that this preventable condition remains associated with catastrophic intracranial hemorrhage and complex medicolegal concerns when prophylactic vitamin K is missed or refused [9]. These reports are consistent with the present case, in which missed prophylaxis, exclusive breastfeeding, severe anemia, prolonged coagulation parameters, and subdural hemorrhage formed a typical pattern of severe late VKDB. However, unlike many reported cases with fatal or disabling outcomes, our patient showed early clinical improvement following prompt vitamin K administration, plasma replacement, transfusion support, and intensive care management.

The diagnosis of VKDB is primarily clinical and laboratory based. Characteristic findings include prolonged prothrombin time and activated partial thromboplastin time in the presence of normal platelet counts and fibrinogen levels. Rapid normalization of coagulation parameters after vitamin K administration is considered highly supportive of the diagnosis and helps distinguish VKDB from inherited coagulation disorders and other causes of acquired coagulopathy [4, 7]. In our patient, significant improvement in coagulation parameters and cessation of bleeding occurred within hours of treatment, further confirming the diagnosis.

Prompt treatment is essential to minimize mortality and neurological complications. Management typically includes vitamin K replacement together with blood product support when significant bleeding or intracranial hemorrhage is present. Fresh frozen plasma rapidly replenishes deficient coagulation factors, while packed red blood cell transfusion corrects severe anemia and improves tissue oxygen delivery. Our patient received intravenous vitamin K, fresh frozen plasma, and packed red blood cell transfusions, resulting in rapid correction of coagulopathy and favorable clinical recovery [5, 8].

The most important lesson from this case is that late VKDB is almost entirely preventable. A single intramuscular dose of vitamin K administered shortly after birth has been shown to dramatically reduce the incidence of both classic and late VKDB and remains the most effective preventive strategy [10, 11]. Despite clear international recommendations, missed prophylaxis continues to occur in settings where home deliveries are common or access to neonatal healthcare is limited. Strengthening awareness among healthcare professionals and parents regarding the importance of neonatal vitamin K administration is essential to prevent avoidable cases of intracranial hemorrhage and infant mortality [11, 12, 13].

This report has some limitations. Neurological follow‐up was limited to 1 week after discharge, and delayed neurodevelopmental sequelae may not be detectable during such a short follow‐up period. Longer‐term developmental surveillance would be necessary to confirm the absence of later motor, cognitive, visual, or seizure‐related complications after intracranial hemorrhage and hypoxic–ischemic cerebral injury.

## 6 Conclusion

Late vitamin K deficiency bleeding remains a preventable yet potentially fatal cause of intracranial hemorrhage in infancy, particularly among exclusively breastfed infants who do not receive vitamin K prophylaxis at birth. This case highlights the importance of early recognition of VKDB in infants presenting with seizures, pallor, or bleeding manifestations, especially in low‐resource settings and home‐delivery contexts. Prompt administration of vitamin K and supportive management can result in favorable outcomes. Universal neonatal vitamin K prophylaxis and parental education are essential to prevent avoidable morbidity and mortality associated with this life‐threatening condition.

## Author Contributions

**Fardowsa Hassan Ahmed:** data curation, investigation. **Mohamed Nur Ali:** conceptualization, methodology. **Ibrahim Mohamed Hirsi:** software, data curation. **Said Abdirahman Ahmed:** writing – original draft, project administration, methodology. **Farah Abdullahi Ismail:** investigation, resources. **Farah Ali Ahmed:** investigation, visualization. **Yasir Khalif Ali:** conceptualization, data curation. **Mohamed Mukhtar Mohamed:** supervision, writing – original draft, writing – review and editing.

## Funding

The authors have nothing to report.

## Ethics Statement

Mogadishu Somali Türkiye Training and Research Hospital (MSTH) does not require institutional review board (IRB) approval for case reports.

## Consent

Written and signed informed consent was obtained from the parents of the patient for the publication of case details.

## Data Availability

The data that support the findings of this study are available in Mogadishu Somali Turkey, Recep Tayyip Erdogan Training and Research Hospital information system. Data are however allowed to the authors upon reasonable request and with permission of the education and research committee.
